# Biomarkers and factors in small cell lung cancer patients treated with immune checkpoint inhibitors: A meta‐analysis

**DOI:** 10.1002/cam4.5800

**Published:** 2023-05-10

**Authors:** Lin‐Lu Li, Cheng‐Feng Yu, Hong‐Ting Xie, Zheng Chen, Bo‐Hui Jia, Fei‐Yu Xie, Ya‐Fang Cai, Peng Xue, Shi‐Jie Zhu

**Affiliations:** ^1^ Department of Oncology Wangjing Hospital, China Academy of Chinese Medical Sciences 100102 Beijing China; ^2^ Graduate School Beijing University of Chinese Medicine 100029 Beijing China; ^3^ Beijing Sihui West District Hospital 100082 Beijing China

**Keywords:** biomarkers, immune checkpoint inhibitors, meta‐analysis, small cell lung cancer

## Abstract

**Objective:**

The aim of this meta‐analysis was to summarize the available results of immunotherapy predictors for small cell lung cancer (SCLC) and to provide evidence‐based information for their potential predictive value of efficacy.

**Methods:**

We searched PubMed, EMBASE, Web of Science, The Cochrane Library, and ClinicalTrials (from January 1, 1975 to November 1, 2021). The hazard ratios (HR) and its 95% confidence intervals (CIs) and tumor response rate of the included studies were extracted.

**Results:**

Eleven studies were eventually included and the pooled results showed that programmed cell death ligand 1 (PD‐L1) positive: objective response rate (ORR) (relative risk [RR] = 1.39, 95% CI [0.48, 4.03], *p* = 0.54), with high heterogeneity (*p* = 0.05, *I* ^2^ = 56%); disease control rate [DCR] (RR = 1.31, 95% CI [0.04, 38.57], *p* = 0.88), with high heterogeneity (*p* = 0.04, *I* ^2^ = 75%); overall survival (OS) (HR = 0.89, 95% CI [0.74, 1.07], *p* = 0.22); and progression‐free survival (PFS) (HR = 0.83, 95% CI [0.59, 1.16], *p* = 0.27), with high heterogeneity (*p* = 0.005, *I* ^2^ = 73.1%). TMB‐High (TMB‐H): OS (HR = 0.86, 95% CI [0.74, 1.00], *p* = 0.05); PFS (HR = 0.71, 95% CI [0.6, 0.85], *p* < 0.001). Lactate dehydrogenase (LDH) >upper limit of normal (ULN): OS (HR = 0.95, 95% CI [0.81, 1.11], *p* = 0.511). Asian patients: OS (HR = 0.87, 95% CI [0.72, 1.04], *p* = 0.135); White/Non‐Asian patients: OS (HR = 0.83, 95% CI [0.76, 0.90], *p* < 0.001). Liver metastasis patients: OS (HR = 0.93, 95% CI [0.83, 1.05], *p* = 0.229); PFS (HR = 0.84, 95% CI [0.67, 1.06], *p* = 0.141). Central nervous system (CNS) metastasis patients: OS (HR = 0.91, 95% CI [0.71, 1.17], *p* = 0.474); PFS (HR = 1.03, 95% CI [0.66, 1.60], *p* = 0.903).

**Conclusion:**

The available research results do not support the recommendation of PD‐L1 positive and TMB‐H as predictors for the application of immune checkpoint inhibitors (ICIs) in SCLC patients. LDH, baseline liver metastasis and CNS metastasis may be used as markers/influencing factors for predicting the efficacy of ICIs in SCLC patients. Non‐Asian SCLC patients had better efficacy with ICIs in our results.

## 1 INTRODUCTION

Small cell lung cancer (SCLC) is a pathological subtype of lung cancer with rapid doubling time, strong aggressiveness, and early widespread metastasis, accounting for 13%–15% of all lung cancer cases.^1^ About 60%–70% of SCLC patients are already in extensive disease (ED) when they were diagnosed,^2^ surgery is typically not recommended. For decades, the standard first‐line treatment for ED‐SCLC has been etoposide in combination with cisplatin or carboplatin.^3^ Although most patients have a high response rate to first‐line chemotherapy (about 70%–80%), many patients relapse within a few months due to chemoresistance,^4^ resulting in a median progression‐free survival (PFS) of 5–6 months and overall survival (OS) of 9–10 months.^3^

In recent years, immunotherapy has become an active area of research and a new direction in the treatment of SCLC. Immune checkpoint inhibitors (ICIs) are essentially humanized monoclonal antibodies that target programmed cell death protein 1 (PD‐1), programmed cell death ligand 1 (PD‐L1), or cytotoxic T lymphocyte‐associated antigen 4 (CTLA‐4) (CD152) to activate T cells and promote the immune system to recognize and attack cancer cells.^5^ The results of IMpower133, a large clinical study of ED‐SCLC, showed that patients in the combination atezolizumab treatment group had a median OS extension of 2 months compared to placebo plus chemotherapy (carboplatin or cisplatin (CP) with etoposide (ET)), with 21.0% and 34.0% of patients surviving in the placebo plus CP/ET arm and atezolizumab plus CP/ET arms, respectively, at month 18.^6^ Unfortunately, not all SCLC patients achieve better clinical benefit with ICIs in addition to chemotherapy.^6^, ^7^, ^8^ Therefore, there are enormous challenges for oncologists in applying ICIs and optimizing results. Searching for predictive markers or influencing factors related to the efficacy of ICIs is important for treatment selection, appropriate clinical management, and reduction of treatment costs.

Many potential biomarkers/influencing factors related to the efficacy of ICIs have been proposed, with PD‐L1 and tumor mutation burden (TMB) being the most frequently studied. PD‐L1 is a ligand for the programmed cell death 1 (PD‐1) receptor.^9^ One of the immune escape mechanisms of tumor cells is the interaction of PD‐1 with PD‐L1 to inhibit T‐cell activation, allowing tumor cells to escape immune surveillance.^10^ It seems logical that the expression of PD‐L1 correlates with the efficacy of ICIs.^11^ Therefore, it has been proposed that high PD‐L1 expression in patients with SCLC is associated with higher response and prolonged patient survival.^12^ However, many patients with high PD‐L1 expression do not achieve the expected antitumor response, while patients with low PD‐L1 expression may still have clinical benefit.^13^ TMB is defined as the total number of somatic mutations, base substitutions, deletions detected, or gene insertions per million bases.^14^ TMB‐High (TMB‐H) increases the number and type of neoantigens produced by tumor cells, and the immune system has a greater chance of recognizing and killing tumor cells.^15^ Therefore, TMB has been proposed as a new biomarker to evaluate the efficacy of ICIs and the prognosis of survival.^16^ However, there are also findings suggesting that TMB does not seem to be a valid marker for the prognosis of cancer patients.^17^ In addition, other influential factors/biomarkers that have been proposed to be associated with tumor prognosis or immunotherapy efficacy include, serum lactate dehydrogenase (LDH),^18^, ^19^ ethnic differences,^20^, ^21^ and metastasis status,^22^, ^23^, ^24^ etc. Likewise, the predictive role of these biomarkers/influencing factors on the efficacy of ICIs in SCLC patients remains to be further explored. In view of the many biomarkers/influencing factors proposed above, and the different outcomes presented by the same biomarker, we conducted a meta‐analysis to summarize trial data from different study outcomes based on the available clinical data, and discuss the biomarkers/influencing factors related to the efficacy of SCLC patients.

## 2 MATERIALS AND METHODS

### 2.1 Search strategy

This meta‐analysis was performed following the PRISMA guidelines according to the Preferred Reporting Items for Systematic Reviews and Meta‐Analyses Protocols (PRISMA‐P) statement. In addition, this meta‐analysis was registered on the PROSPERO database (PROSPERO ID: CRD42022291538). Ethics approval is not required, as this study involved published studies.

Two reviewers (LLL and YCF) independently searched the following databases from January 1, 1975 to November 1, 2021: PubMed, EMBASE, Web of Science, and The Cochrane Library. Unpublished materials searched through the following databases from January 1, 1975 to November 1, 2021: ClinicalTrials. The search strategies in the electronic databases are listed in Table S1.

### 2.2 Inclusion and exclusion criteria

Two reviewers (LLL and YCF) independently selected articles. Disagreements or uncertainties were resolved by the investigators (XHT and CZ).

#### 2.2.1 Inclusion criteria

1. Patients with pathologically confirmed SCLC;
2. Prospective research;
3. ICI therapy was applied;
4. At least one biomarker of PD‐L1, TMB, LDH, liver metastasis, central nervous system (CNS) metastasis.
5. Studies published in English.

#### 2.2.2 Exclusion criteria

1. Cell experiments, animal research, reviews, summary, case reports, commentaries, letters;
2. Studies with insufficient data;
3. Studies with less than 10 patients;
4. Keep articles with the most recent available data from multiple publications of the same study.

### 2.3 Data extraction

Two reviewers (LLL and YCF) independently extracted data using a predesigned data extraction form. The results were compared by the investigators (XHT and CZ), and resolved any disagreements or uncertainties. Extracted details include study characteristics (research name/first author, latest year of publication, type of study), enrollment period, interventions (types of treatment), and results. We collected hazard ratios (HR) and 95% confidence intervals (CIs) to assess OS and PFS; and response rates or number of people per item to assess objective response rate (ORR) and disease control rate (DCR).

### 2.4 Outcome definition

1. Overall survival (OS)
2. Progression‐free survival (PFS)
3. Objective response rate (ORR)

ORR=CR+PR

Complete response (CR), partial response (PR), stable disease (SD), progressive disease (PD).^25^

(4) DCR

DCR=CR+PR+SD

### 2.5 Risk of bias assessment

Two reviewers (LLL and XHT) independently assessed the risk of bias for all candidate studies. Disagreements were determined by the investigators (JBH and CYF). Randomized controlled trials (RCTs) were evaluated with Cochrane Collaboration's tools, and single‐arm or multi‐arm nonrandomized trials used the methodological index for nonrandomized studies (MINORS).^26^

We evaluated RCTs through the following items and each item was classified as high, low, or unclear: random sequence generation, allocation concealment, blinding of participants and personnel, blinding of outcome assessment, incomplete outcome data, selective outcome reporting, and other bias. In the MINORS, there are 12 items: 1–8 items: A clearly stated aim, inclusion of consecutive patients, prospective collection of data, endpoints appropriate to the study aim, unbiased assessment of endpoint, follow‐up period appropriate to the major endpoint, loss to follow‐up no more than 5%, prospective calculation of the sample size. 9–12 items: A gold standard intervention in the control group, contemporary groups, baseline equivalence of groups, and adequate statistical analyses. The first 8 items were used to evaluate single‐arm studies and all 12 items were used to evaluate comparative studies. Each item was scored as 0 (not reported), 1 (reported but inadequate), or 2 (reported and adequate).

### 2.6 Statistical analysis

Statistical analyses were performed using Review manager 5.3 software and Stata 14.0. HR and 95% CI were pooled to evaluate the OS or PFS. Dichotomous variables were expressed as relative risk (RR) to evaluate ORR or DCR. Heterogeneity between studies was assessed by the Cochran's Q test. The fixed effects model was used when heterogeneity was acceptable (*p* > 0.10, *I* ^2^ < 50%) and the random effects model was used when heterogeneity was significant (*p* < 0.10, *I* ^2^ > 50%). The heterogeneities were explored by using subgroup and sensitivity analyses. Begg's and Egger's tests were used to determine publication bias.

## 3 RESULTS

### 3.1 Literature search

**FIGURE 1:**
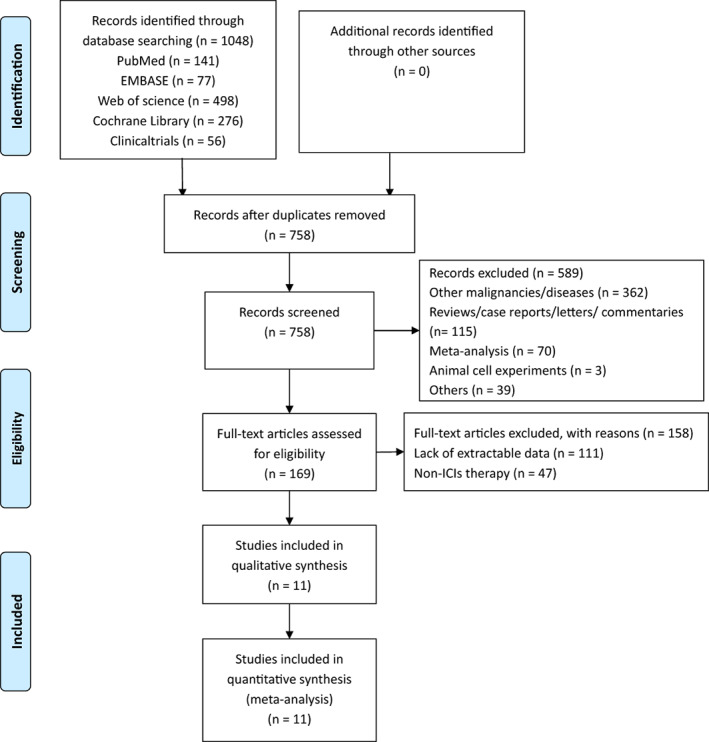
The screening process.

A total of 992 relevant citations and 56 trials were searched through the database. Two hundred ninety duplicate literatures were excluded, and 589 papers that did not meet the writing requirements were excluded based on their abstracts. We conducted a complete literature reading of 169 literatures, and 11 studies were included. The screening process is presented in Figure 1.

### 3.2 Study characteristics

A total of 11 studies with clinical data from 3730 SCLC patients (total number of controlled trials and number of non‐comparative trials applying ICIs) were included.^6^, ^8^, ^27^, ^28^, ^29^, ^30^, ^31^, ^32^, ^33^, ^34^, ^35^, ^36^ Seven studies were randomized studies^6^, ^8^, ^27^, ^31^, ^33^, ^34^, ^35^, ^36^ and the others were non‐randomized, single‐arm or multi‐arm studies. Relevant markers reported included PD‐L1, TMB, LDH, race, liver metastasis, CNS metastasis. Details are shown in Tables 1 and 2.

**TABLE 1:** Characteristics of the included studies.

Author/Study	Registration No.	Enrollment period	Study design	Phase	Country	ICIs Arms	Controls	Dosing of ICIs Arms	Endpoints
Jean‐LouisPujol et al./IFCT‐1603	NCT03059667	2017.3–2017.12	Open‐label randomized noncomparative	II	France	Atezolizumab	Chemotherapy	1200 mg every 3 weeks until progression or unacceptable toxicity	Response rate, OS, PFS, Adverse events, Quality of life
Byoung Chul Cho et al.	NCT03179436	2018.1.15–2019.6.3	A multicenter, open‐label, non‐randomized	I	53 sites	Quavonlimab + pembrolizumab	/	Quavonlimab 75 mg every 6 weeks + pembrolizumab 200 mg every 3 weeks, up to 17 administrations of quavonlimab and 35 administrations of pembrolizumab (~2 years)	Safety, tolerability, ORR, PFS, OS
Hiroaki Akamatsu et al.	NCT03253068	2017.11–2019.10	Open‐label, multi‐institutional, single‐arm	II	Japan	Pembrolizumab + Amrubicin	/	Pembrolizumab 200 mg every 3 weeks on day 1 until disease progression, unacceptable toxicity or up to 2 years + Amrubicin(40 mg/m^2^) every 3 weeks on days 1 to 3 until disease progression or unacceptable toxicity.	ORR, PFS, OS, DOR, safety
Anish Thomas et al.	NCT02484404	2016.4–2018.6	Open‐label, single‐arm	II	NG	Durvalumab + Olaparib	/	Durvalumab 1500 mg every 4 weeks, and olaparib, 300 mg twice a day	ORR, DOR, PFS, OS, safety
CheckMate 032	NCT01928394	2013.11–2016.11	Multicenter, open‐label, two‐stage, multi‐arm	I/II	23 sites, 6 countries	Nivolumab + ipilimumab	/	Nivolumab plus ipilimumab (1 mg/kg plus 1 mg/kg, 1 mg/kg plus 3 mg/kg, or 3 mg/kg plus 1 mg/kg, intravenously) every 3 weeks for four cycles, followed by nivolumab 3 mg/kg every 2 weeks	OS, PFS, ORR, DOR, safety
						Nivolumab	/	Nivolumab alone 3 mg/kg intravenously every 2 weeks until disease progression or unacceptable	OS, PFS, ORR, DOR, safety
KEYNOTE‐158	NCT02628067	/	Open‐label multicohort	II	28 study locations	Pembrolizumab	/	Pembrolizumab 200 mg every 3 weeks was administered for up to 2 years or until documented disease progression, unacceptable toxicity, intercurrent illness preventing further study treatment, or withdrawal	OS, PFS, ORR, DOR, safety and tolerability
CheckMate 331	NCT02481830	2015.8.28–2017.4.24	International, open‐label, randomized trial	III	142 sites across 24 countries	Nivolumab	Chemotherapy (topotecan or amrubicin)	Nivolumab (240 mg) every 2 weeks	OS, PFS, ORR, DOR, safety
IMpower133	NCT02763579	2016.6–2017.5	Multinational, double‐blind, randomized, placebo‐controlled trial	I/III	106 sites, 21 countries	Atezolizumab + CP/ET	Placebo + CP/ET	Carboplatin 5 mg/mL·min on day 1 of each cycle + Eoposide 100 mg/m^2^ on days 1 through 3 of each cycle + Atezolizumab 1200 mg on day 1 of each cycle	OS, PFS, ORR, DOR, safety
CheckMate 451	NCT02538666	2015.10–2018.1	Randomized, double‐blind, three‐arm	III	168 sites, 32 countries	Nivolumab + ipilimumab	Placebo	Nivolumab (1 mg/kg plus ipilimumab 3 mg/kg once every 3 weeks for 12 weeks followed by nivolumab 240 mg once every 2 weeks)	OS, PFS, ORR, DOR, safety
						Nivolumab	Placebo	Nivolumab (240 mg once every 2 weeks)	
CASPIAN	NCT03043872	2017.3–2018.5	Open‐label, sponsor‐blind, randomized, controlled	III	209 cancer treatment centres, 23 countries	Durvalumab + tremelimumab + EP	EP	Durvalumab 1500 mg, tremelimumab 75 mg every 3 weeks, maintenance durvalumab 1500 mg every 4 weeks, until disease progression per investigator assessment, unacceptable toxicity, or other discontinuation criteria	OS, PFS, ORR, overall survival at 18 months, progression‐free survival at 6 months and 12 months, and safety
						Durvalumab + EP	EP	Durvalumab 1500 mg every 3 weeks, maintenance durvalumab 1500 mg every 4 week, until disease progression per investigator assessment, unacceptable toxicity, or other discontinuation criteria	
KEYNOTE‐604	NCT03066778	2017.5–2018.7	Randomized, double‐blind, placebo‐controlled phase III trial	III	140 sites in 18 countries	Pembrolizumab + EP*	Placebo + EP*	Pembrolizumab 200 mg every 3 week once every 3 weeks for 35 cycles or until disease progression, intolerable toxicity, or physician or participant decision	PFS, OS, ORR, DOR, Safety

Abbreviations: CP/ET, carboplatin plus etoposide; EP, etoposide plus either cisplatin or carboplatin; EP*, etoposide and platinum; NG, not given.

**TABLE 2:** Predictor characteristics of the included studies.

Author/Study	ICIs Arms	Outcome	Year	Biomarkers	Sample Source	Cut‐off value	Enrollment people (Total)			Experimental group (ICIs Arms)		
							Sample size	Positive	Negative	Sample size	Positive	Negative
Jean‐LouisPujol et al./IFCT‐1603	Atezolizumab	DCR	2019	PD‐L1	Tumor	TC0:PD‐L1 < 1%;TC1:1% ≤ PD‐L1 < 5%;TC2: 5% ≤ PD‐L1 < 50%;TC3: PD‐L1 ≥ 50%;IC0:PD‐L1 < 1%; IC1:1% ≤ PD‐L1 < 5%;IC2: 5% ≤ PD‐L1 < 10%;IC3: PD‐L1 ≥ 10%	73	16	39	48	8	28
Byoung Chul Cho et al.	Quavonlimab + pembrolizumab	ORR	2021	PD‐L1	Tumor	TPS≥1% or MIDS≥2	40	14	21	40	14	21
Hiroaki Akamatsu et al.	Pembrolizumab + Amrubicin	ORR	2021	PD‐L1	Tumor	CPS≥1	25	19	6	25	19	6
	Pembrolizumab + Amrubicin					TILs>2+	25	13	12	25	13	12
Anish Thomas et al.	Durvalumab + Olaparib	ORR, DCR	2019	PD‐L1	Tumor	Negative or not	20	8	10	20	8	10
CheckMate 032	Nivolumab + ipilimumab	ORR	2018	PD‐L1	Tumor	Staining of tumor‐cell membranes (at any intensity) was observed at prespecifi ed expression levels (≥1% of tumor cells in a section that included ≥100 evaluable tumor cells)	401	43	239	156	19	90
	Nivolumab									245	24	149
KEYNOTE‐158	Pembrolizumab	ORR	2018	PD‐L1	Tumor	CPS≥1	107	42	50	107	42	50
CheckMate 331	Nivolumab	OS, PFS	2021	PD‐L1	tumor cells and immune cells	CPS≥1	321	146	175	171	78	93
		OS		TMB	Tumor	/	487	312(TMB evaluable)	/	245	155(TMB evaluable)	/
		OS		LDH	Blood	>ULN	569	246	293	284	138	137
		OS		Race	/	Asian/white	569	141	422	284	70	211
		OS		brain metastases	/	Yes/No	569	96	473	284	50	234
		OS		Liver metastases	/	Yes/No	569	205	363	284	97	187
IMpower133	Atezolizumab	OS, PFS, ORR	2021	PD‐L1	Tumor tissue	TC/area occupied of IC**≥**1%	403	72	65	201	36	28
		OS, PFS	2021, 2018	TMB	Blood	≥10	403	212	139	201	102	71
						≥16	403	80	271	201	40	133
		OS, PFS	2021, 2018	Brain metastasis		Yes/No	403	35	368	201	17	184
		OS, PFS	2021, 2018	Liver metastasis		Yes/No	403	149	254	201	/	/
CheckMate 451	Nivolumab + ipilimumab	OS, PFS	2021	PD‐L1	Tumor	CPS≥1%	834	163	191	279	52	64
		OS, PFS		TMB	Tumor	≥10	834	276	304	279	90	102
						≥13	834	191	389	279	61	131
		OS		Race	/	Asian/Non‐Asian	834	185	648	279	58	220
		OS		LDH	/	>ULN	834	204	620	279	66	210
		OS		Liver metastasis	/	Yes/No	834	325	509	279	110	169
	Nivolumab	OS, PFS		PD‐L1	Tumor	CPS≥1%	834	163	191	280	55	69
		OS, PFS		TMB	Tumor	≥10	834	276	304	280	98	98
						≥13	834	191	389	280	71	125
		OS		Race	/	Asian/Non‐Asian	834	185	648	280	58	222
		OS		LDH	/	>ULN	834	204	620	280	69	207
		OS		Liver metastasis	/	Yes/No	834	325	509	280	106	174
CASPIAN	Durvalumab + tremelimumab + EP	OS	2021	Brain or CNS metastases	/	Yes/No	805	93	712	268	38	230
				Liver metastases	/	Yes/No	805	329	476	268	117	151
				Race	/	Asian/Non‐Asian	805	125	671	268	47	216
	Durvalumab + EP			Brain or CNS metastases	/	Yes/No	805	93	712	268	28	240
				Liver metastases	/	Yes/No	805	329	476	268	108	160
				Race	/	Asian/Non‐Asian	805	125	671	268	36	231
KEYNOTE‐604	Pembrolizumab + EP	OS	2020	Race	/	East Asia/Not East Asia	453	84	369	228	52	176
		OS		LDH concentration	/	>ULN	453	256	195	228	127	100
		OS, PFS		Brain metastases	/	Yes/No	453	55	398	228	33	195
		OS, PFS		Liver metastases	/	Yes/No	453	187	266	228	95	133
		OS, PFS		PD‐L1	Tumor	CPS≥1%	453	185	175	228	88	97

Abbreviations: CPS, combined positive score; IC, tumor‐infiltrating immune cells; LDH, lactate dehydrogenase; MIDS, mononuclear inflammatory density score; TC, tumor cell; TILs, tumor‐infiltrating lymphocytes; TPS, tumor proportion score; ULN, Upper limit of normal.

Quality assessment tools were used with Cochrane collaboration's tools and MINORS, respectively. Since the CheckMate 032 study^31^ had both random and non‐random cohorts, this study was assessed by applying both evaluation tools, as detailed in Tables 3 and 4.

**TABLE 3:** The Cochrane collaboration's tool assessed the risk of bias in randomized studies.

Author/Study	Random sequence generation	Allocation concealment	Blinding of participants and personnel	Blinding of outcome assessment	Incomplete outcome data	Selective outcome reporting	Other bias
IMpower133	L	L	L	L	L	L	U
CheckMate 451	L	L	L	L	L	L	L
KEYNOTE‐604	L	L	L	L	L	L	L
CheckMate 331	L	H	H	L*	L	L	U
CASPIAN	L	H	H	L*	L	L	L
CheckMate 032	L	H	H	L*	L	L	L
Jean‐LouisPujol et al., 2019	L	H	H	L*	L	L	U

*Note*: L*: Although the study was open‐label, the ICIs response was assessed by a blinded, independent central radiographic review. U: Cross‐over of randomized controlled studies may affect the results, and this item is not discussed.

Abbreviations: H, high; L, low; U, unclear.

**TABLE 4:** The MINORS tool assessed the risk of bias in non‐randomized studies.

Author/Study	A clearly stated aim	Inclusion of consecutive patients	Prospective collection of data	Endpoints appropriate to the aim of the study	Unbiased assessment of the study endpoint	Follow‐up period appropriate to the aim of the study	Loss to follow up less than 5%	Prospective calculation of the study size	An adequate control group	Contemporary groups	Baseline equivalence of groups	Adequate statistical analyses
CheckMate 032	2	2	2	2	2	2	2	2	2	2	2	2
Byoung Chul Cho et al., 2021	2	2	2	2	2	2	0	1*	/	/	/	/
Hiroaki Akamatsu et al., 2021	2	2	2	2	2	2	0	2	/	/	/	/
Anish Thomas et al., 2019	2	2	2	2	2	2	0	2	/	/	/	/
KEYNOTE‐158	2	2	2	2	2	2	0	1*	/	/	/	/

*Note*: The items are scored 0 (not reported), 1 (reported but inadequate), or 2 (reported and adequate). 1*: Not mentioned in the literature, but the ClinicalTrials website states the estimated total number of patients will included but does not elaborate on SCLC.

### 3.3 Meta‐analysis results

#### 3.3.1 PD‐L1

**FIGURE 2:**
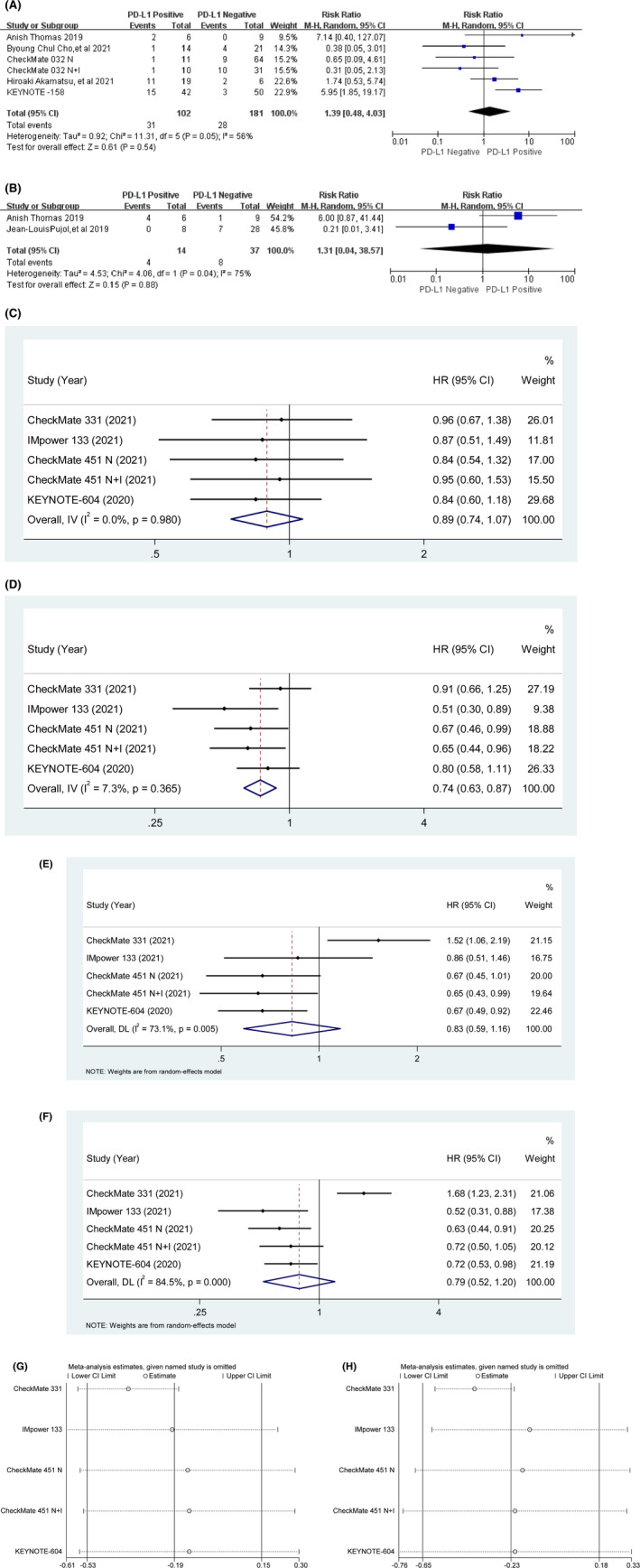
(A) ORR results of ICIs treatment in different PD‐L1 expression status. (B) DCR results of ICIs treatment in different PD‐L1 expression status. (C) OS in PD‐L1‐positive SCLC patients treated with ICIs compared to those with controls. (D) OS in PD‐L1‐negative SCLC patients treated with ICIs compared to those with controls. (E) PFS in PD‐L1‐positive SCLC patients treated with ICIs compared to those with controls. (F) PFS in PD‐L1‐negative SCLC patients treated with ICIs compared to those with controls. (G) Sensitivity analysis of PD‐L1 positive in PFS results. (H) Sensitivity analysis of PD‐L1 negative in PFS results.

ORR results from five studies were extractable. Among SCLC patients who had applied immune checkpoint inhibitors, 102 patients were positive for PD‐L1 expression and 181 patients were negative. The pooled results showed no significant correlation between PD‐L1 expression and benefit from ICIs (RR = 1.39, 95% CI [0.48, 4.03], *p* = 0.54), with high heterogeneity (*p* = 0.05, *I* ^2^ = 56%), a random‐effect model was used (Figure 2A).

The sensitivity analysis was performed by removing the study in turn. After removal of KEYNOTE‐158, the heterogeneity was 22%. KEYNOTE‐158 is an open‐label multicohort study, and the ORR data extracted for SCLC came from the 2018 conference paper^32^ with a large proportion of patients accounted for by the ORR data. Scrutiny of the study's registration information and published papers suggest that this study's bias is small. By comparing other meta‐analyses, the total number of patients with this outcome did not include more than 300 patients, a larger sample size is needed to illustrate the impact of PD‐L1 expression status on the ORR benefit of applying ICIs.

Two studies reported extractable DCR results. Among patients who had immune checkpoint inhibitors applied, 14 patients were positive for PD‐L1 expression and 37 patients were negative. The pooled results showed no significant correlation between PD‐L1 expression and benefit from ICIs (RR = 1.31, 95% CI [0.04, 38.57], *p* = 0.88), with high heterogeneity (*p* = 0.04, *I* ^2^ = 75%), a random‐effect model was used (Figure 2B).

Only two studies had DCR data that could be pooled, and it was difficult to judge the source of heterogeneity of this pooled term by sensitivity analysis.

Four studies explored the association between PD‐L1 expression and OS. The pooled results indicated that the clinical benefit of ICIs was not significant in patients with PD‐L1 positive compared with control treatment (HR = 0.89, 95% CI [0.74, 1.07], *p* = 0.22). With low heterogeneity (*p* = 0.98, *I* ^2^ = 0%), a fixed‐effect model was used. The pooled results indicated that the clinical benefit of ICIs was significant in patients with PD‐L1 negative compared with control treatment (HR = 0.74, 95% CI [0.63, 0.87], *p* < 0.001). With low heterogeneity (*p* = 0.365, *I* ^2^ = 7.3%), a fixed‐effect model was used (Figure 2C,D).

Four studies explored the association between PD‐L1 expression and PFS. The pooled results indicated that the clinical benefit of ICIs was not significant in patients with PD‐L1 positive compared with control treatment (HR = 0.83, 95% CI [0.59, 1.16], *p* = 0.27). With high heterogeneity (*p* = 0.005, *I* ^2^ = 73.1%), a random‐effect model was used. The pooled results indicated that the clinical benefit of ICIs was not significant in patients with PD‐L1 negative compared with control treatment (HR = 0.79, 95% CI [0.52, 1.20], *p* = 0.442). With high heterogeneity (*p* < 0.001, *I* ^2^ = 84.5%), a random‐effect model was used (Figure 2E,F).

The sensitivity analysis was performed by removing the study in turn, when CheckMate 331 was removed, the effect on the confidence interval was greater, suggesting a source of heterogeneity. IMpower 133, CheckMate 451 and KEYNOTE‐604 were randomized, double‐blind trials. This suggests that biases that may arise from subjective reasons in the study should be minimized in the early stages of experimental design (Figure 2G,H).

#### 3.3.2 TMB

**FIGURE 3:**
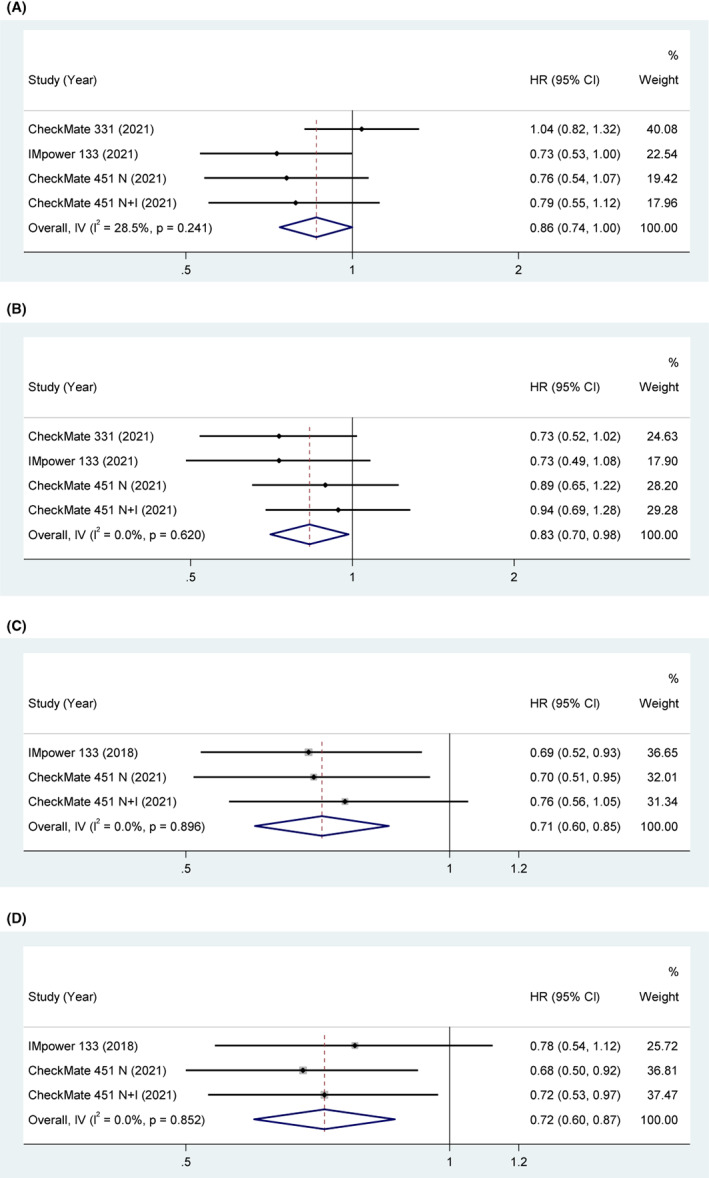
(A) OS in TMB‐H SCLC patients treated with ICIs compared to those with controls. (B) OS in TMB‐L SCLC patients treated with ICIs compared to those with controls. (C) PFS in TMB‐H SCLC patients treated with ICIs compared to those with controls. (D) PFS in TMB‐L SCLC patients treated with ICIs compared to those with controls.

Three studies explored the association between TMB and OS. The pooled results indicated that the clinical benefit of ICIs was significant in patients with TMB‐H compared with control treatment (HR = 0.86, 95% CI [0.74, 1.00], *p* = 0.05). With acceptable heterogeneity (*p* = 0.241, *I* ^2^ = 28.5%), a fixed‐effect model was used. The pooled results indicated that the clinical benefit of ICIs was significant in patients with low TMB (TMB‐L) compared with control treatment (HR = 0.83, 95% CI [0.70, 0.98], *p* = 0.03). With low heterogeneity (*p* = 0.62, *I* ^2^ = 0%), a fixed‐effect model was used (Figure 3A,B).

Two studies explored the association between TMB and PFS. The pooled results indicated that the clinical benefit of ICIs was significant in patients with TMB‐H compared with control treatment (HR = 0.71, 95% CI [0.6, 0.85], *p* < 0.001). With low heterogeneity (*p* = 0.896, *I* ^2^ = 0%), a fixed‐effect model was used. The pooled results indicated that the clinical benefit of ICIs was significant in patients with TMB‐L compared with control treatment (HR = 0.72, 95% CI [0.60, 0.87], *p* < 0.001). With low heterogeneity (*p* = 0.852, *I* ^2^ = 0%), a fixed‐effect model was used in Figure 3C,D.

#### 3.3.3 LDH

**FIGURE 4:**
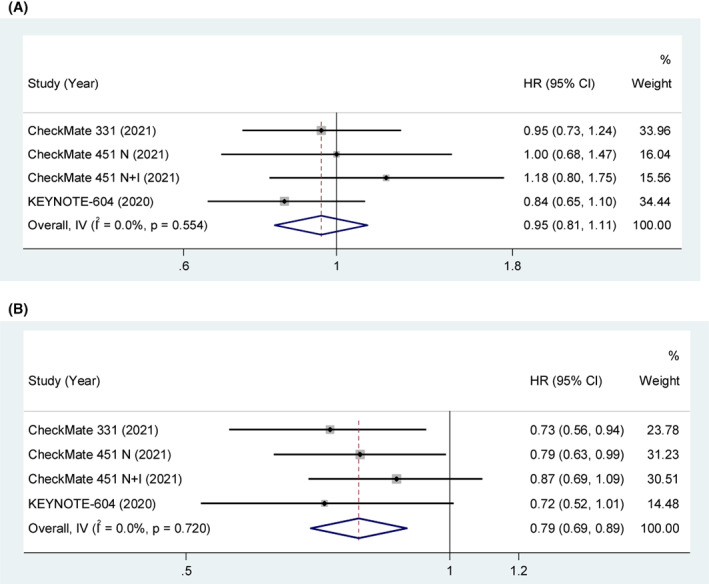
(A) OS in LDH > ULN SCLC patients treated with ICIs compared to those with controls. (B) OS in LDH ≤ ULN SCLC patients treated with ICIs compared to those with controls.

Three studies explored the association between LDH and OS. The pooled results indicated that the clinical benefit of ICIs was not significant in patients with LDH > ULN compared with control treatment (HR = 0.95, 95% CI [0.81, 1.11], *p* = 0.511). With low heterogeneity (*p* = 0.554, *I* ^2^ = 0%), a fixed‐effect model was used. The pooled results indicated that the clinical benefit of ICIs was significant in patients with LDH ≤ ULN compared with control treatment (HR = 0.79, 95% CI [0.69, 0.89], *p* < 0.001). With low heterogeneity (*p* = 0.720, *I* ^2^ = 0%), a fixed‐effect model was used in Figure 4A,B.

#### 3.3.4 Race

**FIGURE 5:**
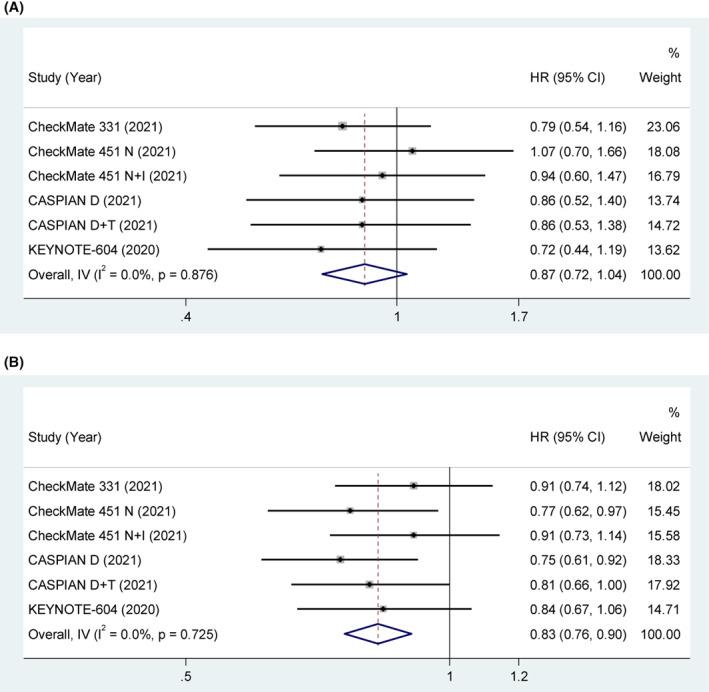
(A) OS in Asian SCLC patients treated with ICIs compared to those with controls. (B) OS in White/Non‐Asian SCLC patients treated with ICIs compared to those with controls.

Four studies explored the association between race and OS. The pooled results indicated that the clinical benefit of ICIs was not significant in Asian patients compared with control treatment (HR = 0.87, 95% CI [0.72, 1.04], *p* = 0.135). With low heterogeneity (*p* = 0.876, *I* ^2^ = 0%), a fixed‐effect model was used. The pooled results indicated that the clinical benefit of ICIs was significant in White/Non‐Asian patients compared with control treatment (HR = 0.83, 95% CI [0.76, 0.90], *p* < 0.001). With low heterogeneity (*p* = 0.725, *I* ^2^ = 0%), a fixed‐effect model was used in Figure 5A,B.

#### 3.3.5 Liver metastasis

**FIGURE 6:**
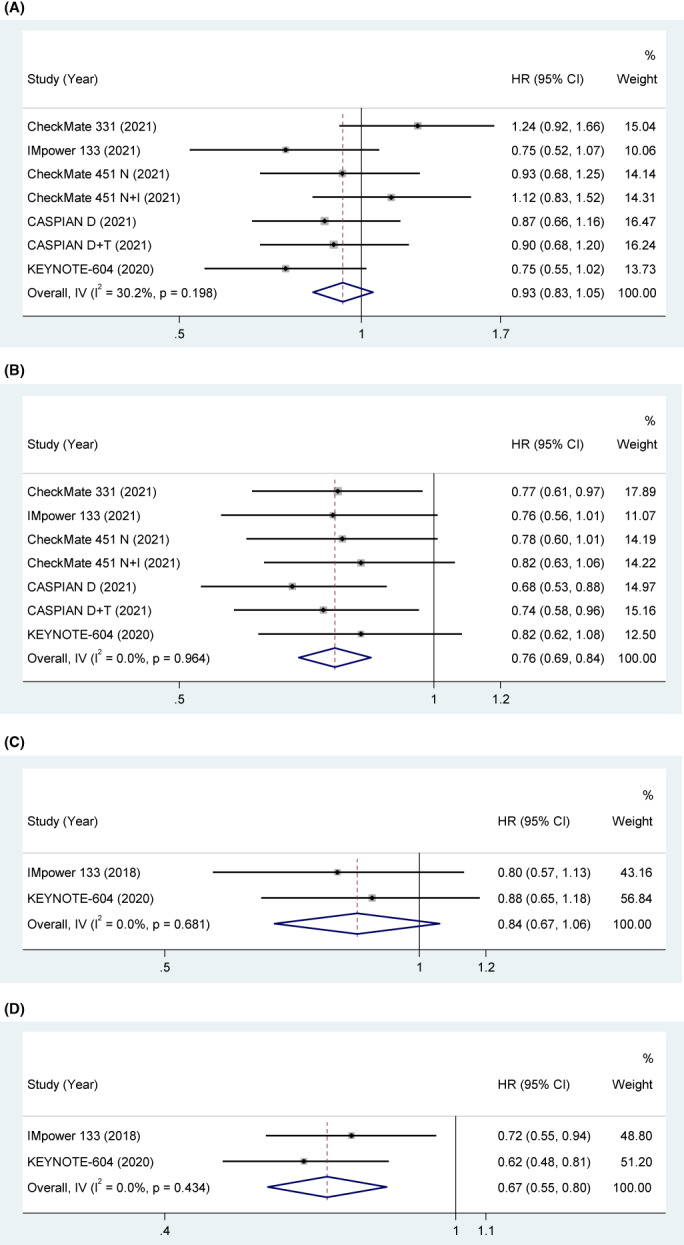
(A) OS in liver metastasis SCLC patients treated with ICIs compared to those with controls. (B) OS in non‐liver metastasis SCLC patients treated with ICIs compared to those with controls. (C) PFS in liver metastasis SCLC patients treated with ICIs compared to those with controls. (D) PFS in non‐liver metastasis SCLC patients treated with ICIs compared to those with controls.

Five studies explored the association between liver metastasis and OS. The pooled results indicated that the clinical benefit of ICIs was not significant in liver metastasis patients compared with control treatment (HR = 0.93, 95% CI [0.83, 1.05], *p* = 0.229). With acceptable heterogeneity (*p* = 0.198, *I* ^2^ = 30.2%), a fixed‐effect model was used. The pooled results indicated that the clinical benefit of ICIs was significant in non‐liver metastasis patients compared with control treatment (HR = 0.76, 95% CI [0.69, 0.84], *p* < 0.001). With low heterogeneity (*p* = 0.964, *I* ^2^ = 0%), a fixed‐effect model was used in Figure 6A,B.

Two studies explored the association between liver metastasis and PFS. The pooled results indicated that the clinical benefit of ICIs was not significant in liver metastasis patients compared with control treatment (HR = 0.84, 95% CI [0.67, 1.06], *p* = 0.141). With low heterogeneity (*p* = 0.681, *I* ^2^ = 0%), a fixed‐effect model was used. The pooled results indicated that the clinical benefit of ICIs was significant in non‐liver metastasis patients compared with control treatment (HR = 0.67, 95% CI [0.55, 0.80], *p* < 0.001). With low heterogeneity (*p* = 0.434, *I* ^2^ = 0%), a fixed‐effect model was used in Figure 6C,D.

#### 3.3.6 CNS metastasis

**FIGURE 7:**
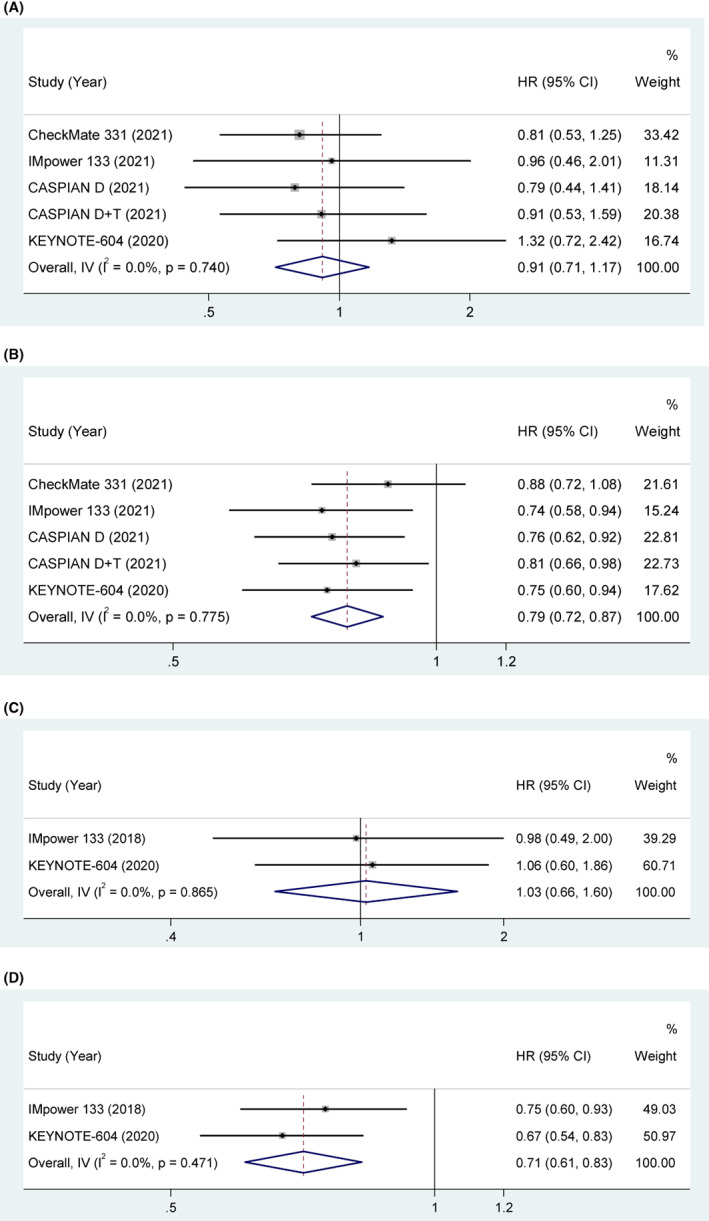
(A) OS in CNS metastasis SCLC patients treated with ICIs compared to those with controls. (B) OS in non‐CNS metastasis SCLC patients treated with ICIs compared to those with controls. (C) PFS in CNS metastasis SCLC patients treated with ICIs compared to those with controls. (D) PFS in non‐CNS metastasis SCLC patients treated with ICIs compared to those with controls.

Four studies explored the association between CNS metastasis and OS. The pooled results indicated that the clinical benefit of ICIs was not significant in CNS metastasis patients compared with those with control treatment (HR = 0.91, 95% CI [0.71, 1.17], *p* = 0.474). With low heterogeneity (*p* = 0.740, *I* ^2^ = 0%), a fixed‐effect model was used. The pooled results indicated that the clinical benefit of ICIs was significant in non‐CNS metastasis patients compared with those with control treatment (HR = 0.79, 95% CI [0.72, 0.87], *p* < 0.001). With low heterogeneity (*p* = 0.775, *I* ^2^ = 0%), a fixed‐effect model was used in Figure 7A,B.

Two studies explored the association between CNS metastasis and PFS. The pooled results indicated that the clinical benefit of ICIs was not significant in CNS metastasis patients compared with control treatment (HR = 1.03, 95% CI [0.66, 1.60], *p* = 0.903). With low heterogeneity (*p* = 0.865, *I* ^2^ = 0%), a fixed‐effect model was used. The pooled results indicated that the clinical benefit of ICIs was significant in non‐CNS metastasis patients compared with those with control treatment (HR = 0.71, 95% CI [0.61, 0.83], *p* < 0.001). With low heterogeneity (*p* = 0.471, *I* ^2^ = 0%), a fixed‐effect model was used in Figure 7C,D.

### 3.4 Publication bias

Since there are not more than 10 studies evaluating PD‐L1, TMB, LDH, race, liver metastasis, and CNS metastasis with clinical benefit, no funnel diagram was drawn.

## 4 DISCUSSION

SCLC is highly bioplastic and heterogeneous,^37^, ^38^ it is difficult to have a unique predictor for ICIs benefit. Considering that no predictor is perfect, in designing this meta‐analysis, we did not limit the search for predictors to PD‐L1 and TMB, two commonly used potential markers. Finally, we also found that LDH, race, liver metastases, and CNS metastases were potential markers/influencing factors for the benefit of ICIs. For the retrieved papers from the same study published at different times, we extracted the most recent available data. For the retrieved trials, we searched the published papers and the updated data of the trial according to the trial registration number and title, and finally extracted the latest data. After a careful and rigorous process, 11 studies with a total of 3730 patients were included in this meta‐analysis. Data extraction and analysis were performed separately according to randomized controlled trials and single‐arm/multi‐arm non‐randomized trials.

Before discussing the results of PD‐L1, we would like to describe the parts of the included studies that need to be highlighted. First, only two of the included studies had extractable DCR data and five studies had extractable ORR data. Three results of Anish Thomas et al.^30^ were controversial (NE; PR unconfirmed; brain only PD, PR elsewhere) and after discussion we excluded them and extracted the results of the other 15 individuals; this exclusion did not increase the heterogeneity of ORR results. In the study of Jean‐LouisPujol et al,^27^ the DCR results in the supplementary file were: PD‐L1‐IC0 (*n* = 29), disease control: 7 (25%), and disease progression: 21 (75%). We discussed and changed the total number of IC0 (*n* = 29) to 28, and this change did not affect the heterogeneity of the DCR results in this study. There were two papers that included the KEYNOTE‐158 study,^32^ and we ultimately selected the conference paper that contained only the KEYNOTE‐158 findings for data extraction, as the other paper combined data from the KEYNOTE‐028 study findings.^7^

Although study has shown that positive expression of PD‐L1 is associated with better OS in SCLC patients,^39^ our meta‐analysis results showed that positive expression of PD‐L1 is difficult to predict the efficacy of ICIs. The results of the study with only ICIs showed that neither ORR nor DCR was significantly higher in the PD‐L1 high expression subgroup than in the PD‐L1 low expression group. Pooled results from controlled studies showed an OS benefit with ICIs/(chemotherapy + ICIs) compared to controls only in patients with PD‐L1‐negative SCLC. There are several possible reasons why PD‐L1 is difficult to use as a predictive biomarker for the efficacy of ICIs. First, PD‐L1 expression is temporally and spatially heterogeneous.^40^ In non‐SCLC studies, PD‐L1 expression appears to be lower in primary tumor samples than in metastases. This suggests that advanced disease may be more likely to express PD‐L1 than earlier stage disease.^41^ Tumor biopsies therefore struggle to capture the complexity of intratumoral heterogeneity and heterogeneous phenotypes in tumor tissue.^42^ Second, the expression of PD‐L1 may be a dynamic process. Because PD‐L1 expression differs between primary and metastatic sites^43^ and can be altered by treatment.^44^ Therefore, a single biopsy may not be sufficient to assess PD‐L1 expression.^45^ It was also been proposed that PD‐L1 expression in circulating tumor cells could be combined for prediction.^46^ In addition, as SCLC has a high frequency of somatic mutations, the combination of PD‐L1 and certain gene mutations may be considered, and such predictive study has been confirmed in NSCLC.^47^ The third is that high PD‐L1 levels predict low disease control rates.^48^ Overexpression of PD‐L1 in many cancers leads to exhausted and unresponsive T cells, promoting immune evasion and tumor progression.^49^ It has also been confirmed that tumor‐derived exosomes expressing PD‐L1 may be an important mediator of tumor immune escape.^50^ Therefore, the non‐standardization of the cut‐off values and positive scores of PD‐L1 expression was proposed as one of the reasons.^51^ The current focus is on the tumor proportion score (TPS), which is the level of PD‐L1 expression in tumor cells. But PD‐L1 is also expressed on immune cells, thus the researchers introduced the concept of a combined positive score (CPS). CPS is the proportion score of the sum of PD‐L1 expressed by tumor cells and tumor‐associated immune cells. Herbst et al.^10^ suggested that response to atezolizumab treatment was significantly associated with high levels of PD‐L1 expression on the surface of TIL, but not on tumor cells. The cut‐off values of PD‐L1 in the studies included in this meta‐analysis were essentially CPS, TPS ≥1% or MIDS ≥2, which are not completely uniform. Therefore, it is critical to score PD‐L1 expression consistently and accurately on tumor cells and immune cells.^52^

In terms of immune response mechanisms, MTB‐H should be associated with better efficacy. TMB‐H indicates the presence of more neoantigens in the tumor microenvironment, promoting an inflammatory response that leads to epigenetic alterations.^53^ The higher somatic mutation rate increases the chance that new tumor epitopes will be developed and presented to immune cells via MHC class I.^54^, ^55^ And higher TMB may be associated with the large number of neoantigens that can be recognized by CD8+ T cells,^56^ which suggests that patients with high TMB may have a higher response to ICIs. But the pooled results of our study^6^, ^8^, ^33^ suggest that SCLC patients who received ICI or ICI + chemotherapy had clinical benefit compared to those with controls, regardless of whether TMB is high or not. One reason for this result is that SCLC is characterized by a high TMB.^57^ Therefore, tumor‐infiltrating lymphocytes (TILs) in the tumor microenvironment that associated with immune responses should be used to predict efficacy, rather than TMB.^58^ The second reason is that the cut‐off value and detection method are still issues that should be considered for TMB as a biomarker.^59^, ^60^ The cut‐off values were not the same across the included studies. Checkmate331^33^ has a cut‐off value of assessable/non‐assessable. The other two studies evaluated with a TMB cut‐off value of 10mut /Mb, 13mut /Mb,^8^ and 16mut /Mb.^6^ Considering that the cut‐off value is specific to the implementation of different studies, we finally combined the studies according to TMB‐H. However, as a biomarker, simply defining a cut‐off value as “high TMB” is not an appropriate predictor of the effect of immunotherapy on each type of tumor.^61^ Finally, existing studies have shown that TMB‐H status does not exclude tumor progression.^62^ Based on these conflicting findings, we concluded that TMB is not an appropriate biomarker for predicting the efficacy of ICIs.^63^

LDH has been proposed as a prognostic efficacy predictive marker for a variety of malignancies, such as B‐cell non‐Hodgkin lymphoma,^64^ non‐SCLC,^65^ and melanoma.^66^ Consistent with the results of existing studies,^67^ the pooled results of this meta‐analysis showed that the clinical benefit was higher in SCLC patients with baseline LDH ≤ ULN. Elevated LDH is also recognized to be associated with increased adverse events of ICIs and leads to shorter OS and PFS.^68^ LDH is essentially an enzyme consisting of a family of six tetrameric isozymes^69^ that catalyze the conversion of pyruvate to lactate and play an important role in glycolysis.^70^ The Warburg effect^71^ suggests that tumor cells derive energy supply from glycolysis even in the absence of hypoxia. Therefore, it has been proposed that LDH reflects the number of biologically active cancer cells,^72^ or more intuitively, the volume of tumor.^73^ Therefore, LDH might be considered as a biomarker for SCLC patients treated with ICIs.

The pooled results of baseline racial data showed that non‐Asian or white SCLC patients benefited more from ICIs relative to controls than those of Asian ethnicity. Similar results were reported in patients with high TMB tumors treated with ICIs and in patients with SCLC treated with ICIs.^74^, ^75^ The first explanation for the ethnically different results comes from differences in the genetic profile. Lin et al.^76^ compared the characteristics of immunological and genomic differences in SCLC between East Asians and Caucasian patients. They found a higher proportion of resting‐type immune cells in the East Asians SCLC cohort than in the Caucasian cohort, such as naive B cells, naive CD4+ T cells, resting NKs, and resting DCs. Studies have reported that VEGFA, TGFB1, and FOXP3 are suppressive mediators and are associated with disrupted recruitment and infiltration of T cells or ICIs resistance.^77^, ^78^, ^79^ Expression levels of these several suppressive mediators were high in the East Asians SCLC cohort.^76^ Second, it cannot be excluded that economic, social, and healthcare access may influence the choice of treatment modality.^80^ Third, differences in sample size may be the cause of this results, with generally smaller numbers of Asians in the included studies.^8^, ^33^, ^35^, ^36^ Although confounding of various factors may lead to differences in the efficacy of ICIs among different races, this result may be expected of further verification because this result may be an evidence‐based basis to support different races to choose different treatment modalities.

The pooled results of this study showed that SCLC patients without liver or CNS metastases at baseline had a longer OS and PFS benefit after treatment with ICIs or ICIs+ chemotherapy compared to those with controls. The occurrence of metastasis is undoubtedly related to poor prognosis. The study has shown that the metastasis of some malignant tumors is related to histological subtypes, liver metastasis, and CNS metastasis are more potential in SCLC.^81^ Among them, the microenvironment of the liver may be more suitable for the survival of SCLC cells with neuroendocrine characteristics, which may be related to the high propensity of SCLC to develop liver metastasis.^82^ The liver is an immunosuppressed organ when liver metastases occur, therefore the efficacy of ICIs can be diminished by a decrease in immune response in both liver metastases and primary hepatocellular carcinoma.^83^, ^84^ Tumeh et al.^85^ observed a significant reduction in the density of CD8 + T cells at the margins of liver metastatic tumors in melanoma patients, which is one of the reasons for the decreased immune response.

The occurrence of brain metastases is considered to be associated with progression, and higher T stage is considered an independent risk factor for the occurrence of CNS metastasis in SCLC.^86^ SCLC is sensitive to chemotherapy and the risk of extracranial progression decreases with intensive chemotherapy; therefore, intracranial progression becomes one of the major types of treatment failure in SCLC.^87^ Zhu et al.^88^ found that a hypoxic tumor microenvironment could activate the HIF‐1α/VEGF pathway, improve the survival rate of lung cancer cells, promote angiogenesis, and resistance to radiotherapy. Clinical research by Yao et al.^89^ also confirmed that there is a significant correlation between serum vascular endothelial growth factor‐A (VEGF‐A) and CNS metastasis and disease progression. Meanwhile elevated LDH has been shown to be associated with tumor progression. Malignant tumors invade and destroy normal tissue cells and can release LDH into the blood.^90^ Combined with the pooled results of LDH, we propose the hypothesis that patients with baseline LDH > ULN and patients with liver or CNS metastases may have worse clinical benefit from ICIs.

Limitations of this study: 1. Large heterogeneity was observed in some of the pooled results. The reasons may be small sample size, single center, and some studies with open labels. 2. There are some unavailable data, such as clinical benefit information for smoking patients. Smoking has been a risk factor for SCLC, but the data we extracted could not be combined; as the subgroups in the CASPIAN study^35^ and in the KEYNOTE‐604 study^36^ were different. 3. Although we carefully screened the retrieved studies, only 11 studies were ultimately included. After combining data from non‐randomized and randomized controlled studies separately, the number of studies per marker subgroup was even smaller. More multicenter, large sample size studies are needed for further exploration.

## 5 CONCLUSION

Based on the results of this meta‐analysis, the available research results do not support the recommendation of PD‐L1‐positive and TMB‐H as predictors for the use of ICIs in SCLC patients. LDH, baseline liver metastasis, and CNS metastasis may be used as biomarkers/influencing factors for predicting the efficacy of ICIs in SCLC patients. Non‐Asian SCLC patients achieved better clinical benefit with ICIs. More multicenter, large‐sample, well‐designed studies are needed to confirm these results.

## AUTHOR CONTRIBUTIONS

**Lin‐Lu Li:** Conceptualization (equal); data curation (equal); formal analysis (equal); methodology (equal); software (equal); validation (equal); writing – original draft (equal). **Cheng‐Feng Yu:** Data curation (equal); formal analysis (equal); writing – original draft (equal); writing – review and editing (equal). **Hong‐Ting Xie:** Data curation (equal); writing – original draft (equal); writing – review and editing (equal). **Zheng Chen:** Data curation (equal); writing – original draft (equal). **Bo‐Hui Jia:** Formal analysis (equal); software (equal); writing – original draft (equal). **Fei‐Yu Xie:** Software (equal); writing – original draft (equal). **Ya‐Fang Cai:** Data curation (equal); writing – original draft (equal). **Peng Xue:** Writing – review and editing (equal). **Shijie Zhu:** Conceptualization (equal); funding acquisition (lead); methodology (equal); project administration (lead); supervision (lead); validation (lead); writing – review and editing (equal).

## CONFLICT OF INTEREST STATEMENT

The authors declare that they have no conflicts of interest regarding the publication of this paper.

## Supporting information


Table S1–S23

## Data Availability

The data used to support the findings of this study were included in the 11 studies.^6^, ^8^, ^27^, ^28^, ^29^, ^30^, ^31^, ^32^, ^33^, ^34^, ^35^, ^36^
