# Supplementary material for: Biomarkers and factors in small cell lung cancer patients treated with immune checkpoint inhibitors: A meta‐analysis
Source: Cancer Med. 2023 May 10;12(10):11211–33. doi: 10.1002/cam4.5800 (PMC10242871; doi:10.1002/cam4.5800)
Supplement: Supplementary file 1 — Table S1–S23 [file CAM4-12-11211-s001.docx]

Table S1. The complete search strategies.

| **Search strategy of English database** | |
| --- | --- |
| #1 | "Small Cell Lung Carcinoma"[Mesh] |
| #2 | ((((((((Small Cell Lung Cancer[Title/Abstract]) OR (Oat Cell Lung Cancer[Title/Abstract])) OR (Carcinoma, Small Cell Lung[Title/Abstract])) OR (Oat Cell Carcinoma of Lung[Title/Abstract])) OR (small cell lung neoplasm[Title/Abstract])) OR (small cell lung tumor[Title/Abstract])) OR (small cell lung malignant[Title/Abstract])) OR (SCLC[Title/Abstract])) OR (Small Cell Cancer Of The Lung[Title/Abstract]) |
| #3 | #1 OR #2 |
| #4 | "Carcinoma, Non-Small-Cell Lung"[Mesh] |
| #5 | (((((((((((((((((Carcinoma, Non Small Cell Lung[Title]) OR (Carcinoma, Non-Small Cell Lung[Title])) OR (Carcinomas, Non-Small-Cell Lung[Title])) OR (Lung Carcinoma, Non-Small-Cell[Title])) OR (Lung Carcinomas, Non-Small-Cell[Title])) OR (Non-Small-Cell Lung Carcinomas[Title])) OR (Non-Small-Cell Lung Carcinoma[Title])) OR (Non Small Cell Lung Carcinoma[Title])) OR (Non-Small Cell Lung Carcinoma[Title])) OR (Non-Small Cell Lung Cancer[Title])) OR (Non small Cell Lung Cancer[Title])) OR (Non-small cell lung neoplasm[Title])) OR (Non small cell lung neoplasm[Title])) OR (Non-small cell lung tumor[Title])) OR (Non small cell lung tumor[Title])) OR (Non-small cell lung malignant[Title])) OR (Non small cell lung malignant[Title])) OR (NSCLC[Title]) |
| #6 | #4 OR #5 |
| #7 | #3 NOT #6 |
| #8 | "Immune Checkpoint Inhibitors"[Mesh] |
| #9 | ((((((((((((((((((((((((((PD-1[Title/Abstract]) OR (PD 1[Title/Abstract])) OR (PD-L1[Title/Abstract])) OR (PD L1[Title/Abstract])) OR (CTLA-4[Title/Abstract])) OR (CTLA 4[Title/Abstract])) OR (immune checkpoint[Title/Abstract])) OR (checkpoint blockade[Title/Abstract])) OR (immune checkpoint blocker[Title/Abstract])) OR (ICI[Title/Abstract])) OR (ICIs[Title/Abstract])) OR (ICB[Title/Abstract])) OR (ICBs[Title/Abstract])) OR (ICP[Title/Abstract])) OR (ICPs[Title/Abstract])) OR (immunotherapy[Title/Abstract])) OR (Nivolumab[Title/Abstract])) OR (Pembrolizumab[Title/Abstract])) OR (Atezolizumab[Title/Abstract])) OR (Avelumab[Title/Abstract])) OR (Durvalumab[Title/Abstract])) OR (Ipilimumab[Title/Abstract])) OR (Tremelimumab[Title/Abstract])) OR (Sintilimab[Title/Abstract])) OR (Toripalimab[Title/Abstract])) OR (Camrelizumab[Title/Abstract])) OR (Tislelizumab[Title/Abstract]) |
| #10 | #8 OR #9 |
| #11 | (((((((((((Progressive-free survival[Title/Abstract]) OR (PFS[Title/Abstract])) OR (Overall survival[Title/Abstract])) OR (OS[Title/Abstract])) OR (Objective response rate[Title/Abstract])) OR (ORR[Title/Abstract])) OR (Disease control rate[Title/Abstract])) OR (DCR[Title/Abstract])) OR (disease-free survival[Title/Abstract])) OR (DFS[Title/Abstract])) OR (relapse free survival[Title/Abstract])) OR (RFS[Title/Abstract]) |
| #12 | ((((((((((tumor mutation burden) OR (tumor mutational burden)) OR (tumor mutation load)) OR (tumor mutational load)) OR (TMB)) OR (PD-L1)) OR (PD L1)) OR (tumor infiltrating lymphocyte)) OR (TIL)) OR (metastases)) OR (biomarker) |
| #13 | #7 AND #10 AND #11 AND #12 |
| #14 | “Humans” |
| #15 | “English” |
| #16 | ("1975/01/01"[Date - Publication] : "2021/11/01"[Date - Publication]) |

Table S2. ORR results of ICIs treatment in different PD-L1 expression status

|  |  | PD-L1 Positive | | PD-L1 Negative | |
| --- | --- | --- | --- | --- | --- |
| Study | Year | Events | Total | Events | Total |
| Anish Thomas 2019 | 2019 | 2 | 6 | 0 | 9 |
| Byoung Chul Cho,et al 2021 | 2021 | 1 | 14 | 4 | 21 |
| CheckMate 032 N | 2018 | 1 | 11 | 9 | 64 |
| CheckMate 032 N+I | 2018 | 1 | 10 | 10 | 31 |
| Hiroaki Akamatsu, et al 2021 | 2021 | 11 | 19 | 2 | 6 |
| KEYNOTE -158 | 2018 | 15 | 42 | 3 | 50 |

Table S3. DCR results of ICIs treatment in different PD-L1 expression status

|  |  | PD-L1 Positive | | PD-L1 Negative | |
| --- | --- | --- | --- | --- | --- |
| Study | Year | Events | Total | Events | Total |
| Jean-LouisPujol,et al | 2019 | 0 | 8 | 7 | 28 |
| Anish Thomas | 2019 | 4 | 6 | 1 | 9 |

Table S4. OS in PD-L1 positive SCLC patients treated with ICIs compared to controls

| Study | Year | HR | 95%CI | |
| --- | --- | --- | --- | --- |
| CheckMate 331 | 2021 | 0.96 | 0.67 | 1.38 |
| IMpower 133 | 2021 | 0.87 | 0.51 | 1.49 |
| CheckMate 451 N | 2021 | 0.84 | 0.54 | 1.32 |
| CheckMate 451 N+I | 2021 | 0.95 | 0.6 | 1.53 |
| KEYNOTE-604 | 2020 | 0.84 | 0.6 | 1.18 |

Table S5. OS in PD-L1 negative SCLC patients treated with ICIs compared to controls

| Study | Year | HR | 95%CI | |
| --- | --- | --- | --- | --- |
| CheckMate 331 | 2021 | 0.91 | 0.66 | 1.25 |
| IMpower 133 | 2021 | 0.51 | 0.3 | 0.89 |
| CheckMate 451 N | 2021 | 0.67 | 0.46 | 0.99 |
| CheckMate 451 N+I | 2021 | 0.65 | 0.44 | 0.96 |
| KEYNOTE-604 | 2020 | 0.8 | 0.58 | 1.11 |

Table S6. PFS in PD-L1 positive SCLC patients treated with ICIs compared to controls

| Study | Year | HR | 95%CI | |
| --- | --- | --- | --- | --- |
| CheckMate 331 | 2021 | 1.52 | 1.06 | 2.19 |
| IMpower 133 | 2021 | 0.86 | 0.51 | 1.46 |
| CheckMate 451 N | 2021 | 0.67 | 0.45 | 1.01 |
| CheckMate 451 N+I | 2021 | 0.65 | 0.43 | 0.99 |
| KEYNOTE-604 | 2020 | 0.67 | 0.49 | 0.92 |

Table S7. PFS in PD-L1 negative SCLC patients treated with ICIs compared to controls

| Study | Year | HR | 95%CI | |
| --- | --- | --- | --- | --- |
| CheckMate 331 | 2021 | 1.68 | 1.23 | 2.31 |
| IMpower 133 | 2021 | 0.52 | 0.31 | 0.88 |
| CheckMate 451 N | 2021 | 0.63 | 0.44 | 0.91 |
| CheckMate 451 N+I | 2021 | 0.72 | 0.50 | 1.05 |
| KEYNOTE-604 | 2020 | 0.72 | 0.53 | 0.98 |

Table S8. OS in TMB-H SCLC patients treated with ICIs compared to controls

| Study | Year | HR | 95%CI | |
| --- | --- | --- | --- | --- |
| CheckMate 331 | 2021 | 1.04 | 0.82 | 1.32 |
| IMpower 133 | 2021 | 0.73 | 0.53 | 1.00 |
| CheckMate 451 N | 2021 | 0.76 | 0.54 | 1.07 |
| CheckMate 451 N+I | 2021 | 0.79 | 0.55 | 1.12 |

Table S9. OS in TMB-L SCLC patients treated with ICIs compared to controls

| Study | Year | HR | 95%CI | |
| --- | --- | --- | --- | --- |
| CheckMate 331 | 2021 | 0.73 | 0.52 | 1.02 |
| IMpower 133 | 2021 | 0.73 | 0.49 | 1.08 |
| CheckMate 451 N | 2021 | 0.89 | 0.65 | 1.22 |
| CheckMate 451 N+I | 2021 | 0.94 | 0.69 | 1.28 |

Table S10. PFS in TMB-H SCLC patients treated with ICIs compared to controls

| Study | Year | HR | 95%CI | |
| --- | --- | --- | --- | --- |
| IMpower 133 | 2018 | 0.69 | 0.52 | 0.93 |
| CheckMate 451 N | 2021 | 0.7 | 0.51 | 0.95 |
| CheckMate 451 N+I | 2021 | 0.76 | 0.56 | 1.05 |

Table S11. PFS in TMB-L SCLC patients treated with ICIs compared to controls

| Study | Year | HR | 95%CI | |
| --- | --- | --- | --- | --- |
| IMpower 133 | 2018 | 0.78 | 0.54 | 1.12 |
| CheckMate 451 N | 2021 | 0.68 | 0.50 | 0.92 |
| CheckMate 451 N+I | 2021 | 0.72 | 0.53 | 0.97 |

Table S12. OS in LDH＞ULN SCLC patients treated with ICIs compared to controls

| Study | Year | HR | 95%CI | |
| --- | --- | --- | --- | --- |
| CheckMate 331 | 2021 | 0.95 | 0.73 | 1.24 |
| CheckMate 451 N | 2021 | 1.00 | 0.68 | 1.47 |
| CheckMate 451 N+I | 2021 | 1.18 | 0.8 | 1.75 |
| KEYNOTE-604 | 2020 | 0.84 | 0.65 | 1.10 |

Table S13. OS in LDH≤ULN SCLC patients treated with ICIs compared to controls

| Study | Year | HR | 95%CI | |
| --- | --- | --- | --- | --- |
| CheckMate 331 | 2021 | 0.73 | 0.56 | 0.94 |
| CheckMate 451 N | 2021 | 0.79 | 0.63 | 0.99 |
| CheckMate 451 N+I | 2021 | 0.87 | 0.69 | 1.09 |
| KEYNOTE-604 | 2020 | 0.72 | 0.52 | 1.01 |

Table S14. OS in Asian SCLC patients treated with ICIs compared to controls

| Study | Year | HR | 95%CI | |
| --- | --- | --- | --- | --- |
| CheckMate 331 | 2021 | 0.79 | 0.54 | 1.16 |
| CheckMate 451 N | 2021 | 1.07 | 0.70 | 1.66 |
| CheckMate 451 N+I | 2021 | 0.94 | 0.60 | 1.47 |
| CASPIAN D | 2021 | 0.86 | 0.52 | 1.40 |
| CASPIAN D+T | 2021 | 0.86 | 0.53 | 1.38 |
| KEYNOTE-604 | 2020 | 0.72 | 0.44 | 1.19 |

Table S15. OS in White/Non-Asian SCLC patients treated with ICIs compared to controls

| Study | Year | HR | 95%CI | |
| --- | --- | --- | --- | --- |
| CheckMate 331 | 2021 | 0.91 | 0.74 | 1.12 |
| CheckMate 451 N | 2021 | 0.77 | 0.62 | 0.97 |
| CheckMate 451 N+I | 2021 | 0.91 | 0.73 | 1.14 |
| CASPIAN D | 2021 | 0.75 | 0.61 | 0.92 |
| CASPIAN D+T | 2021 | 0.81 | 0.66 | 1.00 |
| KEYNOTE-604 | 2020 | 0.84 | 0.67 | 1.06 |

Table S16. OS in liver metastasis SCLC patients treated with ICIs compared to controls

| Study | Year | HR | 95%CI | |
| --- | --- | --- | --- | --- |
| CheckMate 331 | 2021 | 1.24 | 0.92 | 1.66 |
| IMpower 133 | 2021 | 0.75 | 0.52 | 1.07 |
| CheckMate 451 N | 2021 | 0.93 | 0.68 | 1.25 |
| CheckMate 451 N+I | 2021 | 1.12 | 0.83 | 1.52 |
| CASPIAN D | 2021 | 0.87 | 0.66 | 1.16 |
| CASPIAN D+T | 2021 | 0.90 | 0.68 | 1.20 |
| KEYNOTE-604 | 2020 | 0.75 | 0.55 | 1.02 |

Table S17. OS in non-liver metastasis SCLC patients treated with ICIs compared to controls

| Study | Year | HR | 95%CI | |
| --- | --- | --- | --- | --- |
| CheckMate 331 | 2021 | 0.77 | 0.61 | 0.97 |
| IMpower 133 | 2021 | 0.76 | 0.56 | 1.01 |
| CheckMate 451 N | 2021 | 0.78 | 0.60 | 1.01 |
| CheckMate 451 N+I | 2021 | 0.82 | 0.63 | 1.06 |
| CASPIAN D | 2021 | 0.68 | 0.53 | 0.88 |
| CASPIAN D+T | 2021 | 0.74 | 0.58 | 0.96 |
| KEYNOTE-604 | 2020 | 0.82 | 0.62 | 1.08 |

Table S18. PFS in liver metastasis SCLC patients treated with ICIs compared to controls

| Study | Year | HR | 95%CI | |
| --- | --- | --- | --- | --- |
| IMpower 133 | 2018 | 0.8 | 0.57 | 1.13 |
| KEYNOTE-604 | 2020 | 0.88 | 0.65 | 1.18 |

Table S19. PFS in non-liver metastasis SCLC patients treated with ICIs compared to controls

| Study | Year | HR | 95%CI | |
| --- | --- | --- | --- | --- |
| IMpower 133 | 2018 | 0.72 | 0.55 | 0.94 |
| KEYNOTE-604 | 2020 | 0.62 | 0.48 | 0.81 |

Table S20. OS in CNS metastasis SCLC patients treated with ICIs compared to controls

| Study | Year | HR | 95%CI | |
| --- | --- | --- | --- | --- |
| CheckMate 331 | 2021 | 0.81 | 0.53 | 1.25 |
| IMpower 133 | 2021 | 0.96 | 0.46 | 2.01 |
| CASPIAN D | 2021 | 0.79 | 0.44 | 1.41 |
| CASPIAN D+T | 2021 | 0.91 | 0.53 | 1.59 |
| KEYNOTE-604 | 2020 | 1.32 | 0.72 | 2.42 |

Table S21. OS in non-CNS metastasis SCLC patients treated with ICIs compared to controls

| Study | Year | HR | 95%CI | |
| --- | --- | --- | --- | --- |
| CheckMate 331 | 2021 | 0.88 | 0.72 | 1.08 |
| IMpower 133 | 2021 | 0.74 | 0.58 | 0.94 |
| CASPIAN D | 2021 | 0.76 | 0.62 | 0.92 |
| CASPIAN D+T | 2021 | 0.81 | 0.66 | 0.98 |
| KEYNOTE-604 | 2020 | 0.75 | 0.6 | 0.94 |

Table S22. PFS in CNS metastasis SCLC patients treated with ICIs compared to controls

| Study | Year | HR | 95%CI | |
| --- | --- | --- | --- | --- |
| IMpower 133 | 2018 | 0.98 | 0.49 | 2.00 |
| KEYNOTE-604 | 2020 | 1.06 | 0.60 | 1.86 |

Table S23. PFS in non-CNS metastasis SCLC patients treated with ICIs compared to controls

| Study | Year | HR | 95%CI | |
| --- | --- | --- | --- | --- |
| IMpower 133 | 2018 | 0.75 | 0.60 | 0.93 |
| KEYNOTE-604 | 2020 | 0.67 | 0.54 | 0.83 |
